# Single-Frame, Multiple-Frame and Framing Motifs in Genes

**DOI:** 10.3390/life9010018

**Published:** 2019-02-10

**Authors:** Christian J. Michel

**Affiliations:** Theoretical Bioinformatics, ICube, CNRS, University of Strasbourg, 300 Boulevard Sébastien Brant, 67400 Illkirch, France; c.michel@unistra.fr

**Keywords:** single-frame motifs, multiple-frame motifs, framing motifs, gene coding, antiparallel and parallel sequences, early life genes

## Abstract

We study the distribution of new classes of motifs in genes, a research field that has not been investigated to date. A single-frame motif *SF* has no trinucleotide in reading frame (frame 0) that occurs in a shifted frame (frame 1 or 2), e.g., the dicodon *AAACAA* is SF as the trinucleotides *AAA* and *CAA* do not occur in a shifted frame. A motif which is not single-frame SF is multiple-frame MF. Several classes of MF motifs are defined and analysed. The distributions of single-frame SF motifs (associated with an unambiguous trinucleotide decoding in the two $5^{\prime }–3^{\prime }$ and $3^{\prime }–5^{\prime }$ directions) and 5′ unambiguous motifs $5^{\prime }U$ (associated with an unambiguous trinucleotide decoding in the $5^{\prime }–3^{\prime }$ direction only) are analysed without and with constraints. The constraints studied are: initiation and stop codons, periodic codons {AAA,CCC,GGG,TTT}, antiparallel complementarity and parallel complementarity. Taken together, these results suggest that the complementarity property involved in the antiparallel (DNA double helix, RNA stem) and parallel sequences could also be fundamental for coding genes with an unambiguous trinucleotide decoding in the two $5^{\prime }–3^{\prime }$ and $3^{\prime }–5^{\prime }$ directions or the $5^{\prime }–3^{\prime }$ direction only. Furthermore, the single-frame motifs SF with a property of trinucleotide decoding and the framing motifs F (also called circular code motifs; first introduced by Michel (2012)) with a property of reading frame decoding may have been involved in the early life genes to build the modern genetic code and the extant genes. They could have been involved in the stage without anticodon-amino acid interactions or in the Implicated Site Nucleotides (ISN) of RNA interacting with the amino acids. Finally, the SF and MF dipeptides associated with the SF and MF dicodons, respectively, are studied and their importance for biology and the origin of life discussed.

## 1. Introduction

The reading frame coding with trinucleotide sets is a fascinating problem, both theoretical and experimental. Before the discovery of the genetic code, a first code was proposed by Gamow [1] by considering the “key-and-lock” relation between various amino acids, and the rhomb shaped “holes” formed by various nucleotides in the DNA. The proposed model will later prove to be false. A few years later, a class of trinucleotide codes, called comma-free codes, was proposed by Crick et al. [2] for explaining how the reading of a sequence of trinucleotides could code amino acids. In particular, how the correct reading frame can be retrieved and maintained. The four nucleotides {*A*,*C*,*G*,*T*} as well as the 16 dinucleotides {*AA*,…,*TT*} are simple codes which are not appropriate for coding 20 amino acids. However, trinucleotides induce a redundancy in their coding. Thus, Crick et al. [2] conjectured that only 20 trinucleotides among the 64 possible trinucleotides {*AAA*,…,*TTT*} code for the 20 amino acids. Such a bijective code implies that the coding trinucleotides are found only in one frame—the comma-freeness property. The determination of a set of 20 trinucleotides forming a comma-free code has several necessary conditions:

(i) A periodic trinucleotide from the set {*AAA*,*CCC*,*GGG*,*TTT*} must be excluded from such a code. Indeed, the concatenation of *AAA* with itself, for instance, does not allow the (original) reading frame to be retrieved as there are three possible decompositions: …,*AAA*,*AAA*,*AAA*,… (original frame), …*A*,*AAA*,*AAA*,*AA*… and …*AA*,*AAA*,*AAA*,*A*…, the commas showing the adopted decomposition.

(ii) Two non-periodic permuted trinucleotides, i.e., two trinucleotides related by a circular permutation, e.g., *ACG* and *CGA*, must also be excluded from such a code. Indeed, the concatenation of *ACG* with itself, for instance, does not allow the reading frame to be retrieved as there are two possible decompositions: …,*ACG*,*ACG*,*ACG*,… (original frame) and …*A*,*CGA*,*CGA*,*CG*…

Therefore, by excluding the four periodic trinucleotides and by gathering the 60 remaining trinucleotides in 20 classes of three trinucleotides such that, in each class, the three trinucleotides are deduced from each other by a circular permutation, e.g., *ACG*, *CGA* and *GAC*, we see that a comma-free code can contain only one trinucleotide from each class and thus has at most 20 trinucleotides. This trinucleotide number is identical to the amino acid number, thus leading to a code assigning one trinucleotide per amino acid without ambiguity.

In the beginning 1960’s, the discovery that the trinucleotide *TTT*, an excluded trinucleotide in a comma-free code, codes phenylalanine [3], led to the abandonment of the concepts both of a comma-free code [2] and a bijective code as the genetic code is degenerate [4,5,6] with a gene translation in one direction [7].

In 1996, a statistical analysis of occurrence frequencies of the 64 trinucleotides in the three frames of genes of both prokaryotes and eukaryotes showed that the trinucleotides are not uniformly distributed in these three frames [8]. By excluding the four periodic trinucleotides and by assigning each trinucleotide to a preferential frame (frame of its highest occurrence frequency), three subsets $X=X_{0}$, $X_{1}$ and $X_{2}$ of 20 trinucleotides each are found in the frames 0 (reading frame), 1 (frame 0 shifted by one nucleotide in the $5^{\prime }–3^{\prime }$ direction, i.e., to the right) and 2 (frame 0 shifted by two nucleotides in the $5^{\prime }–3^{\prime }$ direction) in genes of both prokaryotes and eukaryotes. The same set X of trinucleotides was identified in average in genes (reading frame) of bacteria, archaea, eukaryotes, plasmids and viruses [9,10]. It contains the 20 following trinucleotides:

(1) X={AAC,AAT,ACC,ATC,ATT,CAG,CTC,CTG,GAA,GAC,GAG,GAT,GCC,GGC,GGT,GTA,GTC,GTT,TAC,TTC}

and codes the 12 following amino acids (three and one letter notation):

(2) X={Ala, Asn, Asp, Gln, Glu, Gly, Ile, Leu, Phe, Thr, Tyr, Val}={A,N,D,Q, E, G, I, L, F, T, Y, V}.

This set X has a strong mathematical property. Indeed, X is a maximal $C^{3}$ self-complementary trinucleotide circular code [8].

The reading frame coding with trinucleotide codes (sets of words) in general terms, i.e., not particularly the genetic code, is a concept which has been studied in Michel [11,12]. We extend it to the motifs (words of codes), a theoretical domain which has been ignored according to our knowledge. Genes (protein coding regions) can be partitioned into two disjoint classes of motifs: the single-frame motifs SF with an unambiguous trinucleotide decoding in the two $5^{\prime }–3^{\prime }$ and $3^{\prime }–5^{\prime }$ directions, and the multiple-frame motifs MF with an ambiguous trinucleotide decoding in at least one direction. A single-frame motif SF has no trinucleotide in reading frame (frame 0) that occurs in a shifted frame (frame 1 or 2). In contrast, a multiple-frame motif MF has at least one trinucleotide in reading frame that occurs in a shifted frame. Some well-known MF motifs are involved in ribosomal frameshifting. The expression of some viral and cellular genes utilizes a -1 programmed ribosomal frameshifting (-1 PRF) [13,14]. This -1 PRF sequence is based on three elements: (i) a slippery motif composed of seven nucleotides at which the change in reading frame occurs; (ii) a spacer motif, usually less than 12 nucleotides; and (iii) a down-stream (3′) stimulatory motif, usually a pseudoknot or a stem-loop. In eukaryotes, the slippery motif fits a consensus heptanucleotide *X,XXY,YYZ*, where *XXX* is any three identical nucleotides, *YYY* represents *AAA* or *TTT*, *Z* represents *A*, *C* or *T*, the commas separating the codons in reading frame [15,16]. The slippery motifs $MF_{1}=A,AAA,AAZ$ and $MF_{2}=T,TTT,TTZ$ are multiple-frame MF. Indeed, the codon *AAA* in reading frame also occurs in the shifted frames 1 and 2 in $MF_{1}$, and similarly with the codon *TTT* in $MF_{2}$. Alternative gene decoding is also possible with +1 programmed ribosomal frameshifting (+1 PRF) which has been particularly observed in *Euplotes* [17]. The identified slippery motif TTT,TAR where R={A,G} is multiple-frame MF. The slippery motifs *AAA*, *CCC*, *GGG* and *TTT* may cause frameshifting during transcription, producing RNAs missing specific nucleotides when compared to template DNA [18,19]. The slippery motifs are not always multiple-frame while stressing that the spacer and the down-stream stimulatory motifs have been very poorly characterized [20] and could also be involved in such a multiple-frame definition. From a theoretical point of view, it is important to extend this concept by increasing the length of such multiple-frame slippery motifs and also by considering their different classes. If the multiple-frame motifs may be involved in ribosomal frameshifting, the single-frame motifs SF and the framing motifs F (also called circular code motifs; first introduced in Michel [21,22]) from the circular codes [8,9,10] (reviews in Michel [23]; Fimmel and Strüngmann [24]) may have been important in early life genes for constructing the modern genetic code and the extant genes (see Discussion).

Several classes of MF motifs are defined: (i) a unidirectional multiple-frame motif $3^{\prime }UMF$ has no trinucleotide in reading frame that occurs in a shifted frame after its reading (i.e., its position in the reading frame) but has at least one trinucleotide in reading frame that occurs in a shifted frame before its reading, e.g., the dicodon *AACACA* is $3^{\prime }UMF$ as the trinucleotides *AAC* and (trivially) *ACA* do not occur in a shifted frame after their reading and as the trinucleotide *ACA* occurs in a shifted frame (precisely frame 1) before its reading; (ii) a unidirectional multiple-frame motif $5^{\prime }UMF$, the opposite, has no trinucleotide in reading frame that occurs in a shifted frame before its reading but has at least one trinucleotide in reading frame that occurs in a shifted frame after its reading, e.g., the dicodon *ACACAA* mirror of *AACACA* is $5^{\prime }UMF$ as the trinucleotides (trivially) *ACA* and *CAA* do not occur in a shifted frame before their reading and as the trinucleotide *ACA* occurs in a shifted frame (precisely frame 2) after its reading; and (iii) a bidirectional multiple-frame motif BMF has at least one trinucleotide in reading frame that occurs in a shifted frame before its reading and has at least one trinucleotide in reading frame that occurs in a shifted frame after its reading (both $3^{\prime }UMF$ and $5^{\prime }UMF$), e.g., the dicodons *AAAAAA* and *ACACAC* are BMF. A 5′ unambiguous motif $5^{\prime }U$, is either a SF motif or a $3^{\prime }UMF$ motif, e.g., the dicodons *AAACAA* (SF motif) and *AACACA* ($3^{\prime }UMF$ motif) belong to the class $5^{\prime }U$.

We will only investigate here the distribution of the single-frame motifs SF associated with an unambiguous trinucleotide decoding in the two $5^{\prime }–3^{\prime }$ and $3^{\prime }–5^{\prime }$ directions, and the 5′ unambiguous motifs $5^{\prime }U$ associated with an unambiguous trinucleotide decoding in the $5^{\prime }–3^{\prime }$ direction only, i.e., a less restrictive class of motifs. The distributions of SF and $5^{\prime }U$ motifs will be analysed without and with constraints. The constraints studied are: (i) with initiation and stop codons; (ii) without periodic codons {AAA,CCC,GGG,TTT}; (iii) with antiparallel complementarity; and (iv) with parallel complementarity.

We will also investigate the particular case of motifs made up of two codons, i.e., the dicodons. The definitions of SF and MF dicodons will thus identify two new classes of dipeptides, the SF and MF dipeptides. The SF dipeptides are coded by dicodons with an unambiguous trinucleotide decoding, in contrast to the MF dipeptides which are coded by dicodons with an ambiguous trinucleotide decoding. The concept of SF and MF dipeptides might be of predictive value to studies of prebiotic metabolites [25]. Peptide evolution on the primitive earth is an active and exciting field of research with cyclic dipeptides [26] and selective formation of *SerHis* dipeptide via phosphorus activation [27,28].

## 2. Method

### 2.1. Recall of Biological Definitions

**Notation** **1.** *Let us denotes the nucleotide 4-letter alphabet* B={A,C,G,T} *where* A *stands for adenine,* C *stands for cytosine,* G *stands for guanine and* T *stands for thymine. The trinucleotide set over* B *is denoted by* $B^{3}=\left\{AAA,\ldots ,TTT\right\}$ *. The set of non-empty words (words, respectively) over* B *is denoted by* $B^{+}$ *(* $B^{*}$ *, respectively).*

**Definition** **1.** *According to the complementary property of the DNA double helix, the nucleotide complementarity map*C:B→B*is defined by*C(A)=T*,*C(C)=G*,*C(G)=C*,*C(T)=A. *According to the complementary and antiparallel properties of the DNA double helix, the trinucleotide antiparallel complementarity map*$C:B^{3}\to B^{3}$*is defined by*$C\left(l_{0}l_{1}l_{2}\right)=C\left(l_{2}\right)C\left(l_{1}\right)C\left(l_{0}\right)$*for all*$l_{0},l_{1},l_{2}\in B$. *The trinucleotide parallel complementarity map*$D:B^{3}\to B^{3}$*is defined by*$D\left(l_{0}l_{1}l_{2}\right)=C\left(l_{0}\right)C\left(l_{1}\right)C\left(l_{2}\right)$*for all*$l_{0},l_{1},l_{2}\in B$.

**Example** **1.** C(ACG)=CGT*and*D(ACG)=TGC.

### 2.2. Recall of Circular Code Definitions

**Definition** **2.** *A set*$S\subseteq \;B^{+}$*is a code if, for each*$x_{1},\ldots ,x_{n},y_{1},\ldots ,y_{m}\in S$*,*n,m≥1*, the condition*$x_{1}\cdots x_{n}=y_{1}\cdots y_{m}$*implies*n=m*and*$x_{i}=y_{i}$*for*i=1,…,n.

**Definition** **3.** *Any non-empty subset of the code* $B^{3}$ *is a code and called trinucleotide code.*

**Definition** **4.** *A trinucleotide code*$X\subseteq B^{3}$*is circular if, for each*$x_{1},\ldots ,x_{n},y_{1},\ldots ,y_{m}\in X$*,*n,m≥1*,*$r\in B^{*}$,$s\in B^{+}$*, the conditions*$sx_{2}\cdots x_{n}r=y_{1}\cdots y_{m}$*and*$x_{1}=rs$*imply*n=m*,*r=ε*(empty word) and*$x_{i}=y_{i}$*for*i=1,…,n.

We briefly recall the proof used here to determine whether a code is circular or not, with the most recent and powerful approach which relates an oriented (directed) graph to a trinucleotide code.

**Definition** **5.** [29]*. Let* $X\subseteq B^{3}$ *be a trinucleotide code. The directed graph* G(X)=(V(X),E(X)) *associated with* X *has a finite set of vertices* V(X) *and a finite set of oriented edges* E(X) *(ordered pairs* [v,w] *where* v,w∈X) defined as follows:

$$\{\begin{array}{c} V\left(X\right)=\left\{N_{1},N_{3},N_{1}N_{2},N_{2}N_{3}:\;N_{1}N_{2}N_{3}\in X\right\}\\ E\left(X\right)=\left\{\left[N_{1},N_{2}N_{3}\right],\left[N_{1}N_{2},N_{3}\right]:\;N_{1}N_{2}N_{3}\in X\right\} \end{array}.$$

The theorem below gives a relation between a trinucleotide code which is circular and its associated graph.

**Theorem** **1.** [29]*. Let* $X\subseteq B^{3}$ *be a trinucleotide code. The following statements are equivalent:*

*(i)*  *The code*X*is circular*.
*(ii)*  *The graph*G(X)*is acyclic.*

**Definition** **6.** *Circular code motifs (first introduced by Michel [21,22]), also called here framing motifs* F *, are motifs from the circular codes. They have the capacity to retrieve, maintain and synchronize the reading frame in genes.*

**Example** **2.** *Let a framing motif* $F_{1}=\;$ *...AGGTAATTACCAG... be constructed with the circular code* X *(1) identified in genes of bacteria, archaea, eukaryotes, plasmids and viruses [8,9,10].*

**Figure 1:**
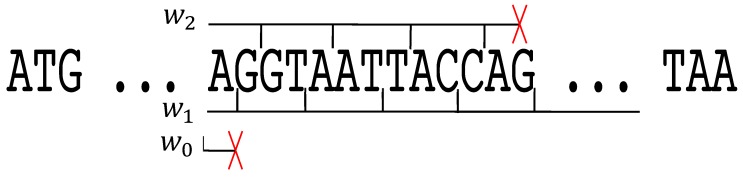
Retrieval of the reading frame of the word w= ...*AGGTAATTACCAG*... constructed with the circular code X (1). Among the three possible factorizations $w_{0}$, $w_{1}$ and $w_{2}$, only one factorization $w_{1}$ into trinucleotides of X is possible leading to ...*A,GGT,AAT,TAC,CAG,*… (the comma showing the reading frame). Thus, the first letter *A* of w is the third letter of a trinucleotide of X and the reading frame of the word is retrieved.

(i) Such a framing motif $F_{1}$ can be obtained as follows. A sequence s of trinucleotides of X is generated and a substring is extracted at any position in this sequence s, i.e., the series of nucleotides on the right and the left of the substring are not considered. Let this substring be $F_{1}$. (ii) This framing motif $F_{1}$ allows the reading frame to be retrieved (Figure 1). We try the three possible decompositions $w_{0}$, $w_{1}$ (shifted by one letter to the right) and $w_{2}$ (shifted by two letters to the right) of $F_{1}$. With $w_{0}$, *AG* is not a prefix of any trinucleotide of X, thus the frame associated with $w_{0}$ is impossible. With $w_{2}$, *AG* is a suffix of *CAG* and *GAG* belonging to X, then *GTA*, *ATT* and *ACC* belong to X, followed by *A* which is a prefix of five trinucleotides of X. Thus at this position, the frame associated with $w_{2}$ is still possible and 2+3×3+1=12 nucleotides are read. The next letter *G* leads to *AG* which is not a prefix of any trinucleotide of X. Thus, a window of 12+1=13 nucleotides demonstrates that the frame associated with $w_{2}$ is impossible. With $w_{1}$, *A* is a suffix of *GAA* and *GTA* belonging to X, then *GGT*, *AAT*, *TAC*, *CAG*, etc., belong to X. Thus, the reading frame of $F_{1}$ is associated with $w_{1}$, i.e., the first letter *A* of w is the 3rd letter of a trinucleotide of X: the reading frame of the sequence s is retrieved: ...*A,GGT,AAT,TAC,CAG,*… (the comma showing the reading frame). (iii) We can prove mathematically that a windows of 13 nucleotides always retrieves the reading frame with the circular code X. Four framing motifs F need a window of 13 nucleotides with the circular code X as they are the four longest ambiguous words of length l=12 nucleotides: $F_{1}=\;$*AGGTAATTACCA*, $F_{2}=\;$*AGGTAATTACCT* (with $w_{2}$, the first two letters *AG* are suffix of *CAG* and *GAG* belonging to X, and the last letter *T* is prefix of *TAC* and *TTC* belonging to X), $F_{3}=\;$*TGGTAATTACCA* (with $w_{2}$, the first two letters *TG* are suffix of *CTG* belonging to X, and the last letter *A* is prefix of five trinucleotides of X) and $F_{4}=\;$*TGGTAATTACCT* (with $w_{2}$, the first two letters *TG* are suffix of *CTG* belonging to X, and the last letter *T* is prefix of *TAC* and *TTC* belonging to X). These four framing motifs F contain the two longest ambiguous words of length l=11 nucleotides starting with a trinucleotide of X, i.e., when the suffixes of X are not considered: *GGTAATTACCA* and *GGTAATTACCT* (see last row in Table 1 in [21]). (iv) It is very important to stress that for all the other framing motifs F of the circular code X, i.e., different from $F_{1}$, $F_{2}$, $F_{3}$ and $F_{4}$, the window for retrieving the reading frame is less than 13 nucleotides (see the growth function of the window as a function of the number of nucleotides in Figure 4 in [21]). It is also very important to recall that any motif of the circular code X is framing, i.e., it has the property of reading frame retrieval.

### 2.3. Definitions of Single-Frame and Multiple-Frame Motifs

**Definition** **7.** *A*n*-motif, also called*n*-codon, is a series of trinucleotides*$t_{i}$*in*$B^{3}$*of trinucleotide length*n*,*i∈{1,…,n}*, which defines the reading frame*f=0*, i.e.,*$t_{1}t_{2}\ldots t_{n}$.

**Definition** **8.** *The shifted frame*f=1*and*f=2*of a*n*-motif is a series of trinucleotides*$t_{i}^{f}$*in*$B^{3}$*of trinucleotide length*n−1*,*i∈{1,…,n−1}*, starting at the 2nd and 3rd nucleotide of*$t_{1}=l_{0}l_{1}l_{2}$*of the*n*-motif, i.e., at*$l_{1}$*(*f=1*) and*$l_{2}$*(*f=2).

**Notation** **2.** *Let* T *be the set of trinucleotides in reading frame* f=0 *of a* n *-motif. Let* $T^{f}$ *be the set of trinucleotides in a shifted frame* f∈{1,2} *of a* n *-motif.*

A single-frame motif SF has no trinucleotide t in reading frame that occurs in a shifted frame, i.e., the trinucleotide decoding is unambiguous in the two $5^{\prime }–3^{\prime }$ and $3^{\prime }–5^{\prime }$ directions. Formally:

**Definition** **9.** *A single-frame*n*-motif*SF*(unambiguous trinucleotide decoding in the two*$5^{\prime }–3^{\prime }$*and*$3^{\prime }–5^{\prime }$*directions) is a*n*-motif such that*$T\cap T^{f}=⊘$*for*f∈{1,2}*, i.e.,*$t_{i}\neq t_{j}^{f}$*for*i∈{1,…,n}*, for*j∈{1,…,n−1}*and for*f∈{1,2}.

**Figure 2:**
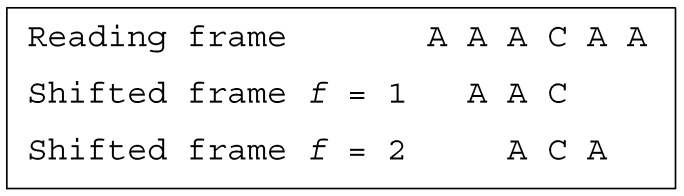
(associated with Example 3). The dicodon *AAACAA* is single-frame SF.

**Example** **3.** *Let the dicodon be AAACAA (*2*-motif). The trinucleotides in reading frame are*$t_{1}=AAA$*and*$t_{2}=CAA$*, leading to the trinucleotide set*T={AAA,CAA}*. The single trinucleotide in the shifted frame 1 is*$t_{1}^{1}=AAC$*, leading to the trinucleotide set*$T^{1}=\left\{AAC\right\}$*. The single trinucleotide in the shifted frame 2 is*$t_{1}^{2}=ACA$*, leading to the trinucleotide set*$T^{2}=\left\{ACA\right\}$*. As*$T\cap T^{1}=⊘$*and*$T\cap T^{2}=⊘$*, AAACAA is a single-frame dicodon*SF*(Figure 2)*.

A multiple-frame motif MF, in contrast to a SF motif, has at least one trinucleotide t in reading frame that occur in a shifted frame f. Formally:

**Definition** **10.** A multiple-frame n -motif MF (ambiguous trinucleotide decoding in at least one direction) is a n -motif such that $T\cap T^{f}\neq ⊘$ for f∈{1,2}, i.e., $\exists i\in \left\{1,\ldots ,n\right\}\wedge \exists j\in \left\{1,\ldots ,n-1\right\}\wedge \exists f\in \left\{1,2\right\}:t_{i}=t_{j}^{f}$.

The unidirectional multiple-frame motifs UMF belong to a class of MF motifs where all the trinucleotides $t^{f}$ in a shifted frame f occur only before $(3^{\prime }UMF$: $3^{\prime }–5^{\prime }$ direction) or only after ($5^{\prime }UMF$: $5^{\prime }–3^{\prime }$ direction) the trinucleotides t in reading frame. Formally:

**Definition** **11.** *A unidirectional multiple-frame*n*-motif*$3^{\prime }UMF$*(ambiguous trinucleotide decoding in the*$3^{\prime }–5^{\prime }$*direction only) is a*MFn*-motif (*$F\cap F^{f}\neq ⊘$*for*f∈{1,2}*) such that the condition*$t_{i}=t_{j}^{f}$*implies*i>j*for*i∈{1,…,n}*, for*j∈{1,…,n−1}*and for*f∈{1,2}.

**Figure 3:**
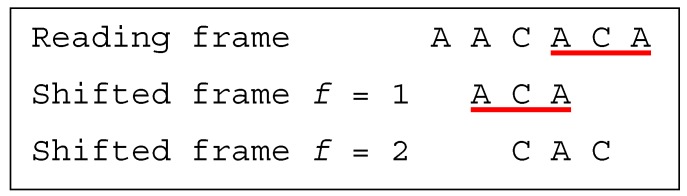
(associated with Example 4). The dicodon *AACACA* is unidirectional multiple-frame 3′UMF.

**Example** **4.** *Let the dicodon be AACACA. The trinucleotides in reading frame are* $t_{1}=AAC$ *and* $t_{2}=ACA$ *, leading to* T={AAC,ACA} *. The single trinucleotide in the shifted frame 1 is* $t_{1}^{1}=ACA$ *, leading to* $T^{1}=\left\{ACA\right\}$ *. The single trinucleotide in the shifted frame 2 is* $t_{1}^{2}=CAC$ *, leading to* $T^{2}=\left\{CAC\right\}$ *. As* $T\cap T^{1}\neq ⊘$ *,* *AACACA* *is a multiple-frame dicodon* MF *. Furthermore, as* $t_{2}=t_{1}^{1}=ACA$ *yields to the inequality* 2>1 *, as* $t_{1}=AAC\neq t_{1}^{1}=ACA$ *and as* $t_{1}=AAC\neq t_{1}^{2}=CAC$ *,* *AACACA* *is a unidirectional multiple-frame dicodon* $3^{\prime }UMF$ *(Figure 3).*

**Definition** **12.** *A unidirectional multiple-frame*n*-motif*$5^{\prime }UMF$*(ambiguous trinucleotide decoding in the*$5^{\prime }–3^{\prime }$*direction only) is a*MFn*-motif (*$F\cap F^{f}\neq ⊘$*for*f∈{1,2}*) such that the condition*$t_{i}=t_{j}^{f}$*implies*i≤j*for*i∈{1,…,n}*, for*j∈{1,…,n−1}*and for*f∈{1,2}.

**Figure 4:**
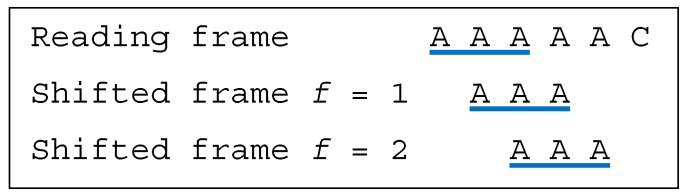
(associated with Example 5). The dicodon *AAAAAC* is unidirectional multiple-frame $5^{\prime }UMF$.

**Example** **5.** *Let the dicodon be AAAAAC. The trinucleotides in reading frame are*$t_{1}=AAA$*and*$t_{2}=AAC$*, leading to*T={AAA,AAC}*. The trinucleotides in the shifted frames 1 and 2 are*$t_{1}^{1}=t_{1}^{2}=AAA$*, leading to the trinucleotide sets*$T^{1}=T^{2}=\left\{AAA\right\}$*. As*$T\cap T^{1}\neq ⊘$*and*$T\cap T^{2}\neq ⊘$*, AAAAAC is a multiple-frame dicodon*MF*. Furthermore, as*$t_{1}=t_{1}^{1}=t_{1}^{2}=AAA$*yields to the two inequalities*1≤1*and as*$t_{2}=AAC\neq t_{1}^{1}=t_{1}^{2}=AAA$*,AAAAAC is a unidirectional multiple-frame dicodon*$5^{\prime }UMF$*(Figure 4)*.

**Figure 5:**
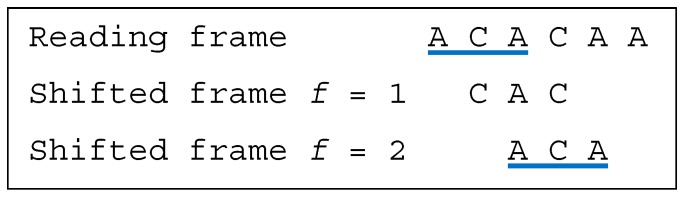
(associated with Example 6). The dicodon *ACACAA* is unidirectional multiple-frame $5^{\prime }UMF$.

**Example** **6.** *Let the dicodon be* *ACACAA* *. The trinucleotides in reading frame are* $t_{1}=ACA$ *and* $t_{2}=CAA$ *, leading to* T={ACA,CAA} *. The single trinucleotide in the shifted frame 1 is* $t_{1}^{1}=CAC$ *, leading to* $T^{1}=\left\{CAC\right\}$ *. The single trinucleotide in the shifted frame 2 is* $t_{1}^{2}=ACA$ *, leading to* $T^{2}=\left\{ACA\right\}$ *. As* $T\cap T^{2}\neq ⊘$ *,* *ACACAA* *is a multiple-frame dicodon* MF *. Furthermore, as* $t_{1}=t_{1}^{2}=ACA$ *yields to the inequality* 1≤1 *, as* $t_{2}=CAA\neq t_{1}^{1}=CAC$ *and as* $t_{2}=CAA\neq t_{1}^{2}=ACA$ *,* *ACACAA* *is a unidirectional multiple-frame dicodon* $5^{\prime }UMF$ *(Figure 5). The reasoning could be immediate by noting that the dicodon ACACAA is mirror of AACACA (compare with Example 4).*

**Definition** **13.** *A bidirectional multiple-frame*n*-motif*BMF*(ambiguous trinucleotide decoding in the two*$5^{\prime }–3^{\prime }$*and*$3^{\prime }–5^{\prime }$*directions) is both a*$5^{\prime }UMF$*and*$3^{\prime }UMF$n-motif.

**Figure 6:**
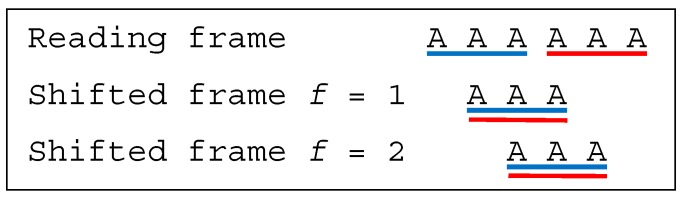
(associated with Example 7). The dicodon *AAAAAA* is bidirectional multiple-frame BMF.

**Example** **7.** *Let the trivial dicodon be AAAAAA. The trinucleotides in reading frame are*$t_{1}=t_{2}=AAA$*, leading to the trinucleotide set*T={AAA}*. The trinucleotides in the shifted frames 1 and 2 are*$t_{1}^{1}=t_{1}^{2}=AAA$*, leading to the trinucleotide sets*$T^{1}=T^{2}=\left\{AAA\right\}$*. As*$T\cap T^{1}\neq ⊘$*and*$T\cap T^{2}\neq ⊘$*, AAAAAA is a multiple-frame dicodon*MF. *Furthermore, as*$t_{1}=t_{1}^{1}=t_{1}^{2}=AAA$*yields to the two inequalities*1≤1*and as*$t_{2}=t_{1}^{1}=t_{1}^{2}=AAA$*yields to the two inequalities*2>1*, AAAAAA is a bidirectional multiple-frame dicodon*BMF*(Figure 6).*

**Figure 7:**
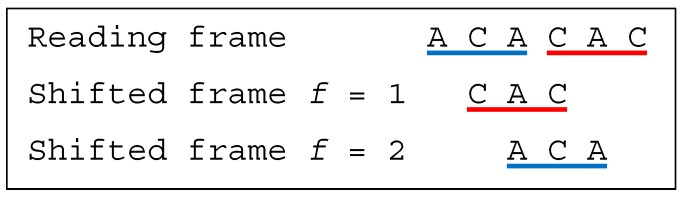
(associated with Example 8). The dicodon *ACACAC* is bidirectional multiple-frame BMF.

**Example** **8.** *Let the dicodon be ACACAC. The trinucleotides in reading frame are*$t_{1}=ACA$*and*$t_{2}=CAC$*, leading to*T={ACA,CAC}*. The single trinucleotide in the shifted frame 1 is*$t_{1}^{1}=CAC$*, leading to*$T^{1}=\left\{CAC\right\}$*. The single trinucleotide in the shifted frame 2 is*$t_{1}^{2}=ACA$*, leading to*$T^{2}=\left\{ACA\right\}$*. As*$T\cap T^{1}\neq ⊘$*and*$T\cap T^{2}\neq ⊘$*, ACACAC is a multiple-frame dicodon*MF. *Furthermore, as*$t_{1}=t_{1}^{2}=ACA$*yields to the inequality*1≤1*and as*$t_{2}=t_{1}^{1}=CAC$*yields to the inequality*2>1*, ACACAC is a bidirectional multiple-frame dicodon*BMF*(Figure 7).*

In this paper, by varying $n\in {\mathbb{N}}^{*}$, we will investigate two distributions: the single-frame n-motifs SF with an unambiguous trinucleotide decoding in the two $5^{\prime }–3^{\prime }$ and $3^{\prime }–5^{\prime }$ directions (see Definition 9), and the 5′ unambiguous n-motifs $5^{\prime }U$ with an unambiguous trinucleotide decoding in the $5^{\prime }–3^{\prime }$ direction only which are defined formally as follows:

**Definition** **14.** *A 5′ unambiguous* n *-motif* $5^{\prime }U$ *(unambiguous trinucleotide decoding in the* $5^{\prime }–3^{\prime }$ *direction only) is either a* SF n *-motif or a* $3^{\prime }UMF$ n *-motif* *, i.e., neither a* $5^{\prime }UMF$ n *-motif nor a* BMF n *-motif.*

**Example** **9.** *The dicodons AAACAA (*SF*motif) andAACACA (*$3^{\prime }UMF$*motif) belong to the class*$5^{\prime }U$.

### 2.4. Occurrence Probabilities of Single-Frame n-Motifs SF and 5′ Unambiguous n-Motifs $5^{\prime }U$

**Definition** **15.** *Let* NbSFM(n) *and* NbMFM(n) *be the numbers of* n *-motifs (* $n\in {\mathbb{N}}^{*}$ *) single-frame* SF *and multiple-frame* MF *, respectively. Let* $Nb5^{\prime }UMFM\left(n\right)$ *,* $Nb3^{\prime }UMFM\left(n\right)$ *and* NbBMFM(n) *be the numbers of multiple-frame* n *-motifs (* $n\in {\mathbb{N}}^{*}$ *) which are unidirectional* $5^{\prime }UMF$ *, unidirectional* $3^{\prime }UMF$ *and bidirectional* BMF *, respectively.*

For $n\in {\mathbb{N}}^{*}$, we have the obvious relations:

$$NbSFM\left(n\right)+NbMFM\left(n\right)=64^{n},\;NbMFM\left(n\right)=Nb5^{\prime }UMFM\left(n\right)+Nb3^{\prime }UMFM\left(n\right)+NbBMFM\left(n\right).$$

For $n\in {\mathbb{N}}^{*}$, the occurrence probability PbSFM(n) of single-frame n-motifs SF will be computed according to

(3) $$PbSFM\left(n\right)=1-\frac{NbMFM\left(n\right)}{64^{n}}.$$

Similarly, for $n\in {\mathbb{N}}^{*}$, the occurrence probability $Pb5^{\prime }UM\left(n\right)$ of 5′ unambiguous n-motifs $5^{\prime }U$ will be computed as follows

(4) $$Pb5^{\prime }UM\left(n\right)=PbSFM\left(n\right)+\frac{Nb3^{\prime }UMFM\left(n\right)}{64^{n}}.$$

**Remark** **1.** *Obviously,*$Pb5^{\prime }UM\left(n\right)>PbSFM\left(n\right)$*whatever*n. *However, it will be interesting to compare these two probability distributions by varying*n.

### 2.5. Single-Frame 1-Motifs

It is a trivial case. Each of the 64 codons (1-motifs, n=1) are obviously single-frame motifs SF, by definition (non-existence of a shifted frame). Thus, the probabilities of SF and $5^{\prime }U$ 1-motifs are equal to $PbSFM\left(1\right)=Pb5^{\prime }UM\left(1\right)=1$.

### 2.6. Single-Frame 2-Motifs

There are $64^{2}=4096$ dicodons (2-motifs, n=2). The complete study of dicodons which are single-frame SF and multiple-frame MF can be done by hand without difficulty. For the convenience of the reader, we give the complete list of MF dicodons: BMF (Definition 13, Table 1), $3^{\prime }UMF$ (Definition 11, Table 2) and $5^{\prime }UMF$ (Definition 12, Table 3).

The probability of SF 2-motifs is equal to $PbSFM\left(2\right)=1-\left(16+2\times 96\right)/64^{2}=0.9492$. The probability of $5^{\prime }U$ 2-motifs is equal to $Pb5^{\prime }UM\left(2\right)=PbSFM\left(2\right)+96/64^{2}=0.9727$.

**Remark** **2.** *For*n≥3*, the*$3^{\prime }UMF$*and*$5^{\prime }UMF$n*-motifs can have two different shifted trinucleotides in the two frames 1 and 2, in contrast to the*2*-motifs (see Table 2 and Table 3). For example, with the tricodon AACAAAACC, the trinucleotides in reading frame are*$t_{1}=AAC$*,*$t_{2}=AAA$*and*$t_{3}=ACC$*leading to*T={AAA,AAC,ACC}*. The trinucleotides in the shifted frame 1 are*$t_{1}^{1}=ACA$*and*$t_{2}^{1}=AAA$*, leading to*$T^{1}=\left\{AAA,ACA\right\}$*. The trinucleotides in the shifted frame 2 are*$t_{1}^{2}=CAA$*and*$t_{2}^{2}=AAC$*, leading to*$T^{2}=\left\{AAC,CAA\right\}$*. As*$T\cap T^{1}\neq ⊘$*and*$T\cap T^{2}\neq ⊘$*, AACAAAACC is a multiple-frame tricodon*MF*. Furthermore, as*$t_{1}=t_{2}^{2}=AAC$*yields to the inequality*1≤2*, as*$t_{2}=t_{2}^{1}=AAA$*yields to the inequality*2≤2*and as*$t_{3}=ACC\notin T^{1}\cup T^{2}$*, AACAAAACC is a unidirectional multiple-frame tricodon*$5^{\prime }UMF$*with two different trinucleotides in the two frames 1 and 2, i.e., AAA in frame 1 and AAC in frame 2*.

### 2.7. Single-Frame n-Motifs

The determination of probability PbSFM(n) of single-frame n-motifs SF for n≥3 (tricodons, tetracodons, etc.) cannot be done by hand. For n∈{3,…,6} (tricodons up to hexacodons), exact values of probability PbSFM(n) can be obtained by computer calculus (see Table 4). For n=6, the computation of SF motifs among the $64^{6}=68,719,476,736$ hexacodons with a parallel program with 8 threads takes about 7 days on a standard PC. For n≥7 (heptacodons, octocodons, etc.), the probability PbSFM(n) is obtained by computer simulation. Simulated values of PbSFM(n) are obtained by generating 1,000,000 random n-motifs for each n. In order to evaluate this approach by computer simulation, simulated values of PbSFM(n) for n∈{2,…,6} are also given in Table 4. Exact and simulated values of PbSFM(n) are identical at $10^{-3}$, demonstrating the reliability of the simulation approach.

The probability $Pb5^{\prime }UM\left(n\right)$ of 5′ unambiguous n-motifs $5^{\prime }U$ for n≥3 is computed similarly.

## 3. Results

### 3.1. Single-Frame Motifs

I first investigated the probability PbSFM(n) (Equation (3)) of single-frame n-motifs SF (Definition 9). The probability PbSFM(1) is equal to 1 (1-motifs, Section 2.5). The probability PbSFM(2) is equal to 94.9% (2-motifs, Section 2.6). The probability PbSFM(n) for n∈{3,…,6} is given in Table 4. The probability PbSFM(n) for n≥7 is obtained by computer simulation (Section 2.7).

**Figure 8:**
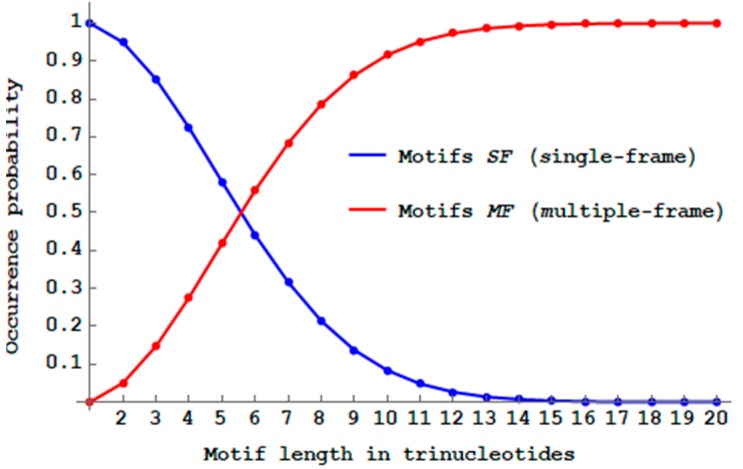
Decreasing probability PbSFM(n) (Equation (3)) of single-frame n -motifs SF (blue curve) and increasing probability 1−PbSFM(n) of multiple-frame n -motifs MF (red curve) by varying the length n between 1 and 20 trinucleotides.

While the proportion of multiple-frame 2-motifs MF (Definition 10) is minimal (5.1%=100%−94.9% for dicodons, Section 2.6), Figure 8 shows that their propagation will drastically reduce the proportion of SF n-motifs when the trinucleotide length n increases. There are almost no more SF motifs with a length of 14 trinucleotides (PbSFM(14)<1%) and the number of MF motifs becomes already higher than the number of SF motifs with a length of six trinucleotides (Figure 8).

Thus, only short genes, i.e., with up to five trinucleotides, have a higher proportion of single-frame motifs compared to the multiple-frame motifs. Thus, primitive translation, without the extant complex ribosome, could only generate short peptides without frameshift errors.

### 3.2. 5′ Unambiguous Motifs

**Figure 9:**
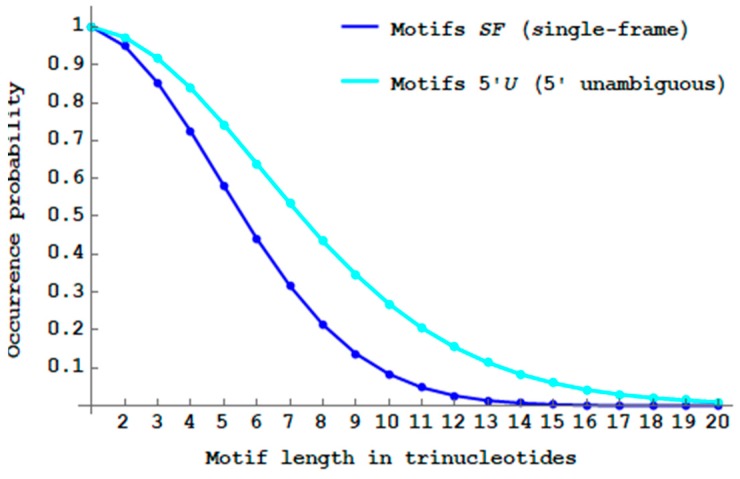
Decreasing probability PbSFM(n) (Equation (3)) of single-frame n -motifs SF (blue curve from Figure 8) and decreasing probability $Pb5^{\prime }UM\left(n\right)$ (Equation (4)) of 5′ unambiguous n -motifs $5^{\prime }U$ (cyan curve) by varying the length n between 1 and 20 trinucleotides.

I then compared the probability PbSFM(n) (Equation (3)) of single-frame n-motifs SF (Definition 9) and the probability $Pb5^{\prime }UM\left(n\right)$ (Equation (4)) of 5′ unambiguous n-motifs $5^{\prime }U$ (Definition 14). Figure 9 shows the decreasing probability $Pb5^{\prime }UM\left(n\right)$ of $5^{\prime }U$ n-motifs when the trinucleotide length n increases. As expected (see Remark 1), its decrease is slower than that of SF n-motifs. There are almost no more $5^{\prime }U$ motifs with a length of 20 trinucleotides ($Pb5^{\prime }UM\left(20\right)<1\%$). Thus with the $5^{\prime }U$ motifs, there is a length increase of 20−14=6 trinucleotides in the trinucleotide decoding. The maximum probability difference $Pb5^{\prime }UM\left(n\right)-PbSFM\left(n\right)$ is 22.0% at length n=8 trinucleotides.

The 5′ unambiguous n-motifs, a less restrictive class of motifs with an unambiguous trinucleotide decoding in the $5^{\prime }–3^{\prime }$ direction only, can generate a slightly longer peptides without frameshift error compared to the single-frame motifs.

I now evaluate the single-frame motifs SF and the 5′ unambiguous motifs $5^{\prime }U$ with constraints.

### 3.3. Single-Frame and 5′ Unambiguous Motifs with Initiation and Stop Codons

**Figure 10:**
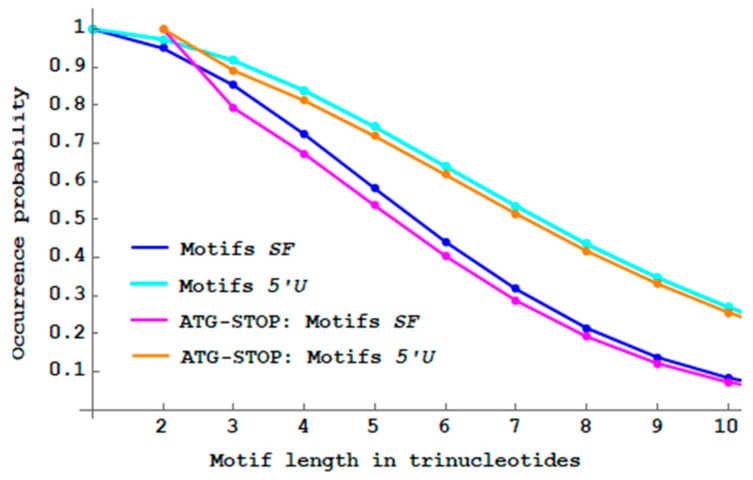
Decreasing probability PbSFM(n) (Equation (3)) of single-frame n -motifs SF (blue curve from Figure 8) and decreasing probability $Pb5^{\prime }UM\left(n\right)$ (Equation (4)) of 5′ unambiguous n -motifs $5^{\prime }U$ (cyan curve from Figure 9) by varying the length n between 1 and 10 trinucleotides. With initiation and stop codons, decreasing probability PbSFM(n) of n -motifs SF (magenta curve) and decreasing probability $Pb5^{\prime }UM\left(n\right)$ of n -motifs $5^{\prime }U$ (orange curve) by varying the length n between 2 and 10 trinucleotides.

The single-frame n-motifs SF and the 5′ unambiguous motifs $5^{\prime }U$ are investigated with an initiation codon *ATG* and a stop codon {TAA,TAG,TGA}. The case n=1 does not exist. For n=2, there are only three dicodons: *ATGTAA*, *ATGTAG* and *ATGTGA* which are all obviously SF. Thus, the probabilities of SF and USF 2-motifs are obviously $PbSFM\left(2\right)=Pb5^{\prime }UM\left(2\right)=1$. Figure 10 shows that the proportions of SF and $5^{\prime }U$ motifs with initiation and stop codons are lower than their respective non-constrained motifs.

Genes with initiation and stop codons do not increase translation fidelity compared to non-constrained genes (according to this approach).

### 3.4. Single-Frame and 5′ Unambiguous Motifs without Periodic Codons

**Figure 11:**
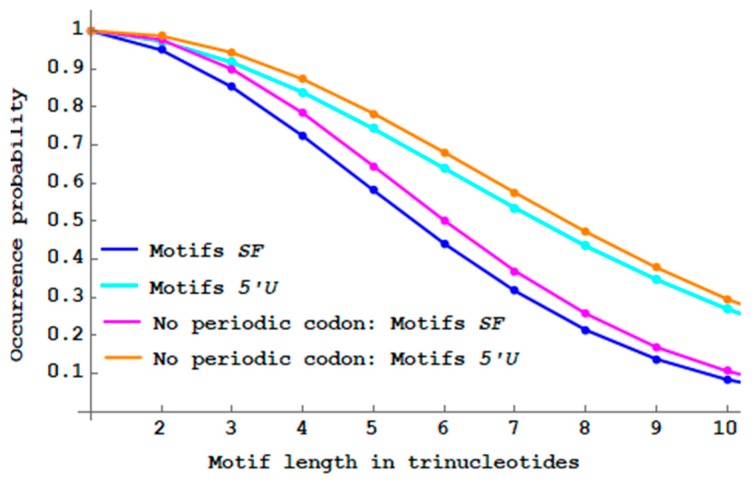
Decreasing probability PbSFM(n) (Equation (3)) of single-frame n -motifs SF (blue curve from Figure 8) and decreasing probability $Pb5^{\prime }UM\left(n\right)$ (Equation (4)) of 5′ unambiguous n -motifs $5^{\prime }U$ (cyan curve from Figure 9) by varying the length n between 1 and 10 trinucleotides. Without periodic codons {AAA,CCC,GGG,TTT}, decreasing probability PbSFM(n) of n -motifs SF (magenta curve) and decreasing probability $Pb5^{\prime }UM\left(n\right)$ of n -motifs $5^{\prime }U$ (orange curve) by varying the length n between 1 and 10 trinucleotides.

The single-frame motifs SF and the 5′ unambiguous motifs $5^{\prime }U$ are now studied without periodic codons {AAA,CCC,GGG,TTT}. As expected, Figure 11 shows that the proportions of SF and $5^{\prime }U$ motifs without periodic codons are higher than their respective non-constrained motifs.

Genes without periodic codons slightly increase frame translation fidelity compared to non-constrained genes (according to this approach).

### 3.5. Single-Frame and 5′ Unambiguous Motifs with Antiparallel Complementarity

**Figure 12:**
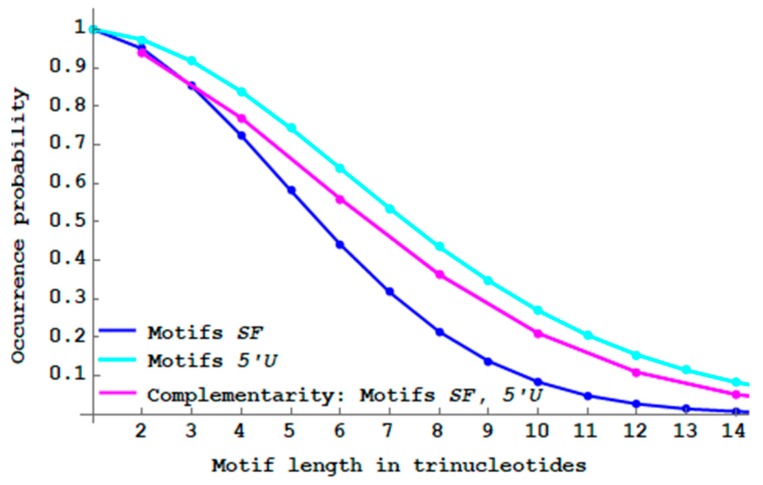
Decreasing probability PbSFM(n) (Equation (3)) of single-frame n -motifs SF (blue curve from Figure 8) and decreasing probability $Pb5^{\prime }UM\left(n\right)$ (Equation (4)) of 5′ unambiguous n -motifs $5^{\prime }U$ (cyan curve from Figure 9) by varying the length n between 1 and 14 trinucleotides. With antiparallel complementarity, decreasing probabilities PbSFM(n) and $Pb5^{\prime }UM\left(n\right)$ of 2n -motifs SF and $5^{\prime }U$ (two identical curves in magenta) by varying the length n between 1 and 7 trinucleotides.

The single-frame 2n-motifs SF and the 5′ unambiguous 2n-motifs $5^{\prime }U$ are now investigated with the following antiparallel complementary sequence: $t_{1}t_{2}\cdots t_{n}C\left(t_{n}\right)\cdots C\left(t_{2}\right)C\left(t_{1}\right)$ where the trinucleotide antiparallel complementarity map C applied to a trinucleotide t is recalled in Definition 1. As an example, if $t_{1}t_{2}t_{3}=ACGTGCAAT$ then the antiparallel complementary sequence studied is ACGTGCAATATTGCACGT. Note that the trinucleotide length of such motifs is even. Classical antiparallel complementary structures are the DNA double helix and the RNA stem. Interesting results are observed. As expected, the two probability curves PbSFM(n) of SF motifs and $Pb5^{\prime }UM\left(n\right)$ of $5^{\prime }U$ motifs with antiparallel complementarity are identical (Figure 12). The proof is based on the following property: if $t_{i}=t_{j}^{f}$ with i>j ($3^{\prime }UMF$ motif) then $C\left(t_{i}\right)=t_{i^{\prime }}=C\left(t_{j}^{f}\right)=t_{j^{\prime }}^{f^{\prime }}$ with $i^{\prime }\leq j^{\prime }$ ($5^{\prime }UMF$ motif) and $f\neq f^{\prime }$. Furthermore, antiparallel complementarity increases the proportion of SF motifs but decreases the proportion of $5^{\prime }U$ motifs, compared to their respective non-constrained motifs.

The “antiparallel complementary” genes have a higher proportion of single-frame motifs compared to the non-complementary genes. Thus, primitive translation associated with a DNA property could generate a greater number of peptides without frameshift errors.

### 3.6. Single-Frame Motifs and 5′ Unambiguous with Parallel Complementarity

**Figure 13:**
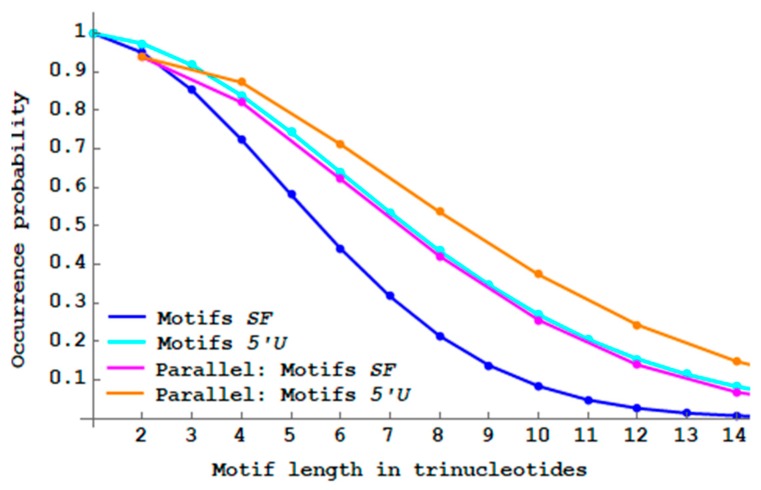
Decreasing probability PbSFM(n) (Equation (3)) of single-frame n -motifs SF (blue curve from Figure 8) and decreasing probability $Pb5^{\prime }UM\left(n\right)$ (Equation (4)) of 5′ unambiguous n -motifs $5^{\prime }U$ (cyan curve from Figure 9) by varying the length n between 1 and 14 trinucleotides. With parallel complementarity, decreasing probability PbSFM(n) of 2n -motifs SF (magenta curve) and decreasing probability $Pb5^{\prime }UM\left(n\right)$ of 2n -motifs $5^{\prime }U$ (orange curve) by varying the length n between 1 and 7 trinucleotides.

The single-frame 2n-motifs SF and the 5′ unambiguous 2n-motifs $5^{\prime }U$ are now analysed with the following parallel complementary sequence: $t_{1}t_{2}\ldots t_{n}D\left(t_{1}\right)D\left(t_{2}\right)\ldots D\left(t_{n}\right)$ where the trinucleotide parallel complementarity map D applied to a trinucleotide t is recalled in Definition 1. As an example, if $t_{1}t_{2}t_{3}=ACGTGCAAT$ then the parallel complementary sequence studied is ACGTGCAATTGCACGTTA. Note that the trinucleotide length of such motifs is also even. Interesting results are also observed. The two probability curves PbSFM(n) of SF motifs with parallel complementarity and $Pb5^{\prime }UM\left(n\right)$ of $5^{\prime }U$ motifs without constraints are superposable (Figure 13). Parallel complementarity increases the proportions of both SF motifs and $5^{\prime }U$ motifs compared to their respective non-constrained motifs.

“Parallel complementary” genes have a slightly higher proportion of single-frame motifs compared to the “antiparallel complementary” genes (compare the magenta curves in Figure 12 and Figure 13). The biological meaning is not yet explained.

### 3.7. Framing Motifs

There are framing motifs F which are single-frame SF or multiple-frame MF.

**Proposition** **1.** *A framing motif*F*can be single-frame*SF.

Proof. Take the following motif m=GAACTCCCGATATGGCTC. The motif m can be generated by the code X={ATA,CCG,CTC,GAA,TGG}. By Theorem 1, it is easy to verify that the graph G(X) is acyclic, and thus X is circular. Furthermore, the set of trinucleotides in reading frame is T=X, the set of trinucleotides in the shifted frame 1 is $T^{1}=\left\{AAC,CGA,GGC,TAT,TCC\right\}$ and the set of trinucleotides in the shifted frame 2 is $T^{2}=\left\{ACT,ATG,CCC,GAT,GCT\right\}$. We have $T\cap T^{1}=⊘$ and $T\cap T^{2}=⊘$. Thus, the motif m is both framing F and single-frame SF.

**Proposition** **2.** *A framing motif*F*can be multiple-frame*MF.

Proof. Take the following motif m=ATTGAGCGAGCCTGTCAG. The motif m can be generated by the code X={ATT,CAG,CGA,GAG,GCC,TGT}. By Theorem 1, it is easy to verify that the graph G(X) is acyclic, and thus X is circular. Furthermore, we have the trinucleotide sets T=X, $T^{1}=\left\{AGC,CCT,GAG,GTC,TTG\right\}$ and $T^{2}=\left\{AGC,CTG,GCG,TCA,TGA\right\}$ leading to $T\cap T^{1}=\left\{GAG\right\}$ and $T\cap T^{2}=⊘$. Thus, the motif m is both framing F and multiple-frame MF, precisely unidirectional multiple-frame $5^{\prime }UMF$.

There are single-frame motifs SF or multiple-frame motifs MF which are not framing F.

**Proposition** **3.** *A single-frame motif*SF*can be non-framing*F.

Proof. Take the following motif m=GACAAATAAGTGGTATGA. The motif m can be generated by the code X={AAA,GAC,GTA,GTG,TAA,TGA}. We have the trinucleotide sets T=X, $T^{1}=\left\{AAG,AAT,ACA,TAT,TGG\right\}$ and $T^{2}=\left\{AGT,ATA,ATG,CAA,GGT\right\}$ leading to $T\cap T^{1}=⊘$ and $T\cap T^{2}=⊘$. However, as X contains the periodic trinucleotide *AAA*, X is not circular. Thus, the motif m is single-frame SF but not framing F.

**Proposition** **4.** *A multiple-frame motif*MF*can be non-framing*F.

Proof. Take the following motif m=GGACCATACATCCGGACT. The motif m can be generated by the code X={ACT,ATC,CCA,CGG,GGA,TAC}. We have the trinucleotide sets T=X, $T^{1}=\left\{ACA,CAT,GAC,GGA,TCC\right\}$ and $T^{2}=\left\{ACC,ATA,CAT,CCG,GAC\right\}$ leading to $T\cap T^{1}=\left\{GGA\right\}$ and $T\cap T^{2}=⊘$. However, as X contains the two permuted trinucleotides *ACT* and *TAC*, X is not circular. Thus, the motif m is multiple-frame MF, precisely unidirectional multiple-frame $5^{\prime }UMF$, but not framing F.

Genes which are both framing F and single-frame SF retrieve the reading frame and code for a unique peptide as the shifted frames would lead to a different peptide product.

### 3.8. A New Class of Theoretical Parameters Relating the Circular Codes and Their Circular Code Motifs

The idea is to define a new class of parameters in order to measure the intensity I(m) of a motif m of a circular code to retrieve the reading frame. Thus, we have to associate information from the circular code theory with information from words (motifs).

In the circular code theory, the most important and the simplest parameter is the length $l_{max}\left(X\right)$ of a longest path (maximal arrow-length of a path) in the associated graph G(X) of a circular code X (see Definition 5). Note that the longest path $l_{max}\left(X\right)$ has a finite length as the graph G(X) is acyclic (Theorem 1). The longest path $l_{max}\left(X\right)$ can classify the circular codes, from the strong comma-free codes with $l_{max}\left(X\right)=1$ and the comma-free codes with $l_{max}\left(X\right)=2$ up to the general circular codes with a maximal longest path $l_{max}\left(X\right)=8$ when $X\subseteq B^{3}$ (i.e., for the trinucleotide circular codes) [29]. It is also related to the reading frame number $n_{X}$ of X, i.e., the number of nucleotides to retrieve the reading frame. This reading frame number $n_{X}$ can also be used to classify the circular codes, from the strong comma-free codes with $n_{X}=2$ nucleotides and the comma-free codes with $n_{X}=3$ nucleotides up to the general circular codes with a maximal number $n_{X}=13$ nucleotides when $X\subseteq B^{3}$ [30]. However, this parameter $n_{X}$ needs to know the structure of the longest path $l_{max}\left(X\right)$ which is one of the four cases: $b_{1}\to d_{1}\to \ldots \to b_{k}$, $b_{1}\to d_{1}\to \ldots \to d_{k}$, $d_{1}\to b_{1}\to \ldots \to b_{k}$ and $d_{1}\to b_{1}\to \ldots \to d_{k}$ where the nucleotide $b_{i}\in B$ and the dinucleotide $d_{i}\in B^{2}$ for any i (see Definition 5). In summary, for the circular codes $X\subseteq B^{3}$, the longest path $l_{max}\left(X\right)$ belongs to the interval $1\leq l_{max}\left(X\right)\leq 8$ and the reading frame number $n_{X}$ belongs to the interval $2\leq n_{X}\leq 13$ nucleotides. The definition of the reading frame number $n_{X}$ can still be generalized to arbitrary sequences, i.e., not entirely consisting of trinucleotides from X [30]. For these two reasons, i.e., the knowledge of the structure of $l_{max}\left(X\right)$ and the generalized definition of $n_{X}$, the parameter $n_{X}$, mentioned here to take date, will not be considered here.

A motif m of a code, circular or not, can be characterized by its length l(m), given here in trinucleotides for convenience, for measuring its expansion; and its cardinality card(T(m)) of the set T(m) (see Notation 2) of trinucleotides (in reading frame f=0) of m for measuring its variety (complexity). In the case of a motif m of a trinucleotide circular code $X\subseteq B^{3}$, 1≤card(T(m))≤20.

It is important to stress the following condition: T(m)⊆X with a trinucleotide circular code $X\subseteq B^{3}$. The case T(m)=X is associated with a trinucleotide circular code X constructed from the motif m.

A simple parameter measuring the expansion intensity $I_{e}\left(m\right)$ of reading frame retrieval of a circular code motif m can be defined as follows:

(5) $$I_{e}\left(m\right)=\frac{l\left(m\right)}{l_{max}\left(X\right)}$$

where l(m), l(m)≥1, is the trinucleotide length of the motif m and $l_{max}\left(X\right)$, $1\leq l_{max}\left(X\right)\leq 8$, is the length of a longest path in the associated graph G(X) of a trinucleotide circular code $X\subseteq B^{3}$. Note that $\frac{1}{8}\leq I_{e}\left(m\right)\leq l\left(m\right)$ and if $l\left(m\right)\geq l_{max}\left(X\right)$ then $1\leq I_{e}\left(m\right)\leq l\left(m\right)$.

A second parameter measuring both the expansion and variety intensity $I_{ev}\left(m\right)$ of a circular code motif m can also be defined as follows:

(6) $$I_{ev}\left(m\right)=\mathrm{card}\left(T\left(m\right)\right)\times I_{e}\left(m\right)$$

where $I_{e}\left(m\right)$ is defined in Equation (5) and card(T(m)), 1≤card(T(m))≤20, is the cardinality of the set T(m) (Notation 2) of trinucleotides (in reading frame f=0) of m. Note that $\frac{1}{8}\leq I_{ev}\left(m\right)\leq 20l\left(m\right)$ and if $l\left(m\right)\geq l_{max}\left(X\right)$ then $1\leq I_{ev}\left(m\right)\leq 20l\left(m\right)$. Thus, for the circular code motifs m of a given trinucleotide length l(m), the intensity $I_{ev}\left(m\right)$ of reading frame retrieval increases according to their cardinality card(T(m)).

For a sequence s containing several circular code motifs m, the formulas (5) and (6) can be expressed as follows:

(7) $$I_{e}\left(s\right)=\underset{m\in s}{\sum }I_{e}\left(m\right)=\frac{\sum_{m\in s}l\left(m\right)}{l_{max}\left(X\right)}$$

with the hypothesis that $l_{max}\left(X\right)$ is identical for the motifs m, a realistic case when the motifs m are obtained from a same studied trinucleotide circular code X, and thus:

(8) $$I_{ev}\left(s\right)=\underset{m\in s}{\sum }I_{ev}\left(m\right)=\frac{\sum_{m\in s}\mathrm{card}\left(T\left(m\right)\right)\times l\left(m\right)}{l_{max}\left(X\right)}.$$

Note also that the formulas $I_{e}\left(s\right)$ and $I_{ev}\left(s\right)$ can also be normalized in order to weight the different lengths of sequences s.

### 3.9. MF Dipeptides

The series of multi-frame motifs MF starts with the dicodons. We will now focus on the MF dipeptides which are two consecutive amino acids coded by the MF dicodons. The 16 bidirectional multiple-frame dicodons BMF (Table 1) code 16 BMF dipeptides according to the universal genetic code (Table 5). They include the four obvious BMF dipeptides *GlyGly* (*GGGGGG*), *LysLys* (*AAAAAA*), *PhePhe* (*TTTTTT*) and *ProPro* (*CCCCCC*). 15 amino acids out of 20 are involved in these 16 BMF dipeptides (Table 6): *Ala*, *Arg*, *Cys*, *Glu*, *Gly*, *His*, *Ile*, *Leu*, *Lys*, *Phe*, *Pro*, *Ser*, *Thr*, *Tyr* and *Val* (except *Asn*, *Asp*, *Gln*, *Met* and *Trp*), each amino acid occurring once in a position of a BMF dipeptide, except *Arg* occurring twice in a position of a BMF dipeptide: ArgAla, ArgGlu, AlaArg and GluArg.

The 96 unidirectional multiple-frame dicodons $3^{\prime }UMF$ (Table 2) code 83 $3^{\prime }UMF$ dipeptides and four pairs (stop codon, amino acid): *TAGArg*, *TAGGly*, *TGAGlu* and *TerLys* where *Ter* can be the two stop codons *TAA* and *TGA* (Table 7). All the 20 amino acids are involved in the 83 $3^{\prime }UMF$ dipeptides (Table 8). All the 20 amino acids occur in the first position of $3^{\prime }UMF$ dipeptides. Five amino acids *Asn*, *Asp*, *Gln, Met* and *Trp* do not occur in their second position which are the five amino acids not involved in the BMF dipeptides. In the 83 $3^{\prime }UMF$ dipeptides, *Pro* and *Gly* are involved 20 and 19 times, respectively, while *Met* and *Trp* only twice and once, respectively.

The 96 unidirectional multiple-frame dicodons $5^{\prime }UMF$ (Table 3) code 40 $5^{\prime }UMF$ dipeptides and three pairs (amino acid, stop codon): *IleTer* where *Ter* can be the two stop codons *TAA* and *TAG*, *PheTer* where *Ter* can be the three stop codons *TAA*, *TAG* and *TGA*, and *ValTGA* (Table 9). All the 20 amino acids are involved in the 40 $5^{\prime }UMF$ dipeptides (Table 10). Five amino acids are *Asn*, *Asp*, *Gln, Met* and *Trp* do not occur in the first position of $5^{\prime }UMF$ dipeptides which are the five amino acids not involved in the BMF dipeptides. All the 20 amino acids occur in their second position. In the 40 $5^{\prime }UMF$ dipeptides, two amino acids *Lys* and *Phe* are involved eight times while *Asn* only once.

The 114=121−4−3 MF dipeptides among 400, i.e., 28.5%, are coded by 208=16+2×96 MF dicodons (BMF, $3^{\prime }UMF$, $5^{\prime }UMF$) among 4096, i.e., 5.1% (Table 11). As a consequence, 286 SF dipeptides, i.e., 71.5%, are coded by 3888 single-frame dicodons SF, i.e., 94.9%. There is also a strong asymmetry between the number of MF dipeptides coded by one direction or other direction: 83 $3^{\prime }UMF$ dipeptides (Table 7) versus 40 $5^{\prime }UMF$ dipeptides (Table 9). This asymmetry may be related to the gene translation in the $5^{\prime }–3^{\prime }$ direction, the $3^{\prime }UMF$ dicodons having an unambiguous trinucleotide decoding in the $5^{\prime }–3^{\prime }$ direction.

Five dipeptides *GlyAla*, *GlyVal*, *PheSer*, *ProLeu* and *ProArg* are the most strongly coded, each by five MF dicodons (Table 12), e.g., *GlyAla* is coded by one $3^{\prime }UMF$ dicodon *GGCGCG* (Table 7), and four $5^{\prime }UMF$ dicodons *GGGGCA, GGGGCC, GGGGCG* and *GGGGCT* (Table 9). The SF and MF dipeptides could have particular spatial structures and biological functions in extant and primitive proteins which remain to be identified.

## 4. Discussion

For the first time to our knowledge, new definitions of motifs in genes are presented. The single-frame motifs SF (unambiguous trinucleotide decoding in the two $5^{\prime }–3^{\prime }$ and $3^{\prime }–5^{\prime }$ directions) and the multiple-frame motifs MF (ambiguous trinucleotide decoding in at least one direction) form a partition of genes. Several classes of MF motifs are defined and analysed: (i) unidirectional multiple-frame motifs 3′UMF (ambiguous trinucleotide decoding in the $3^{\prime }–5^{\prime }$ direction only); (ii) unidirectional multiple-frame motifs $5^{\prime }UMF$ (ambiguous trinucleotide decoding in the $5^{\prime }–3^{\prime }$ direction only); and (iii) bidirectional multiple-frame motifs BMF (ambiguous trinucleotide decoding in the two $5^{\prime }–3^{\prime }$ and $3^{\prime }–5^{\prime }$ directions). The distribution of the single-frame motifs SF and the 5′ unambiguous motifs $5^{\prime }U$ (unambiguous trinucleotide decoding in the $5^{\prime }–3^{\prime }$ direction only) are studied without and with constraints.

The proportion of SF motifs drastically decreases with their trinucleotide length. The SF motifs become absent (<1%) when their length ≥14 trinucleotides and the number of MF motifs becomes already higher than the number of SF motifs when their length ≥6 trinucleotides. As expected, the proportion of $5^{\prime }U$ motifs decreases more slowly than that of SF motifs. The $5^{\prime }U$ motifs become absent (<1%) when their length ≥20 trinucleotides. Thus with the $5^{\prime }U$ motifs, there is a length increase of 20−14=6 trinucleotides in the trinucleotide decoding.

The proportions of SF and $5^{\prime }U$ motifs with initiation and stop codons are lower than their respective non-constrained motifs. In contrasts, their proportions in motifs without periodic codons {AAA,CCC,GGG,TTT} are higher than their respective non-constrained motifs. The proportions of SF and $5^{\prime }U$ motifs with antiparallel complementarity are identical. Antiparallel complementarity increases the proportion of SF motifs but decreases the proportion of $5^{\prime }U$ motifs, compared to their respective non-constrained motifs. The proportions of SF motifs with parallel complementarity and $5^{\prime }U$ motifs without constraints follow a similar distribution. Finally, parallel complementarity increases the proportions of both SF motifs and $5^{\prime }U$ motifs compared to their respective non-constrained motifs. Taken together, these results suggest that the complementarity property involved in the antiparallel (DNA double helix, RNA stem) and parallel sequences could also be fundamental for coding genes with unambiguous trinucleotide decoding, strictly in the two $5^{\prime }–3^{\prime }$ and $3^{\prime }–5^{\prime }$ directions (SF motifs) or conserved in the $5^{\prime }–3^{\prime }$ direction but relaxed-lost in the $3^{\prime }–5$ direction ($5^{\prime }U$ motifs).

The single-frame motifs SF with a property of trinucleotide decoding and the framing motifs F with a property of reading frame decoding could have operated in the primitive soup for constructing the modern genetic code and the extant genes [31]. They could have been involved in the stage without anticodon-amino acid interactions to form peptides from prebiotically amino acids [32]. They could also have been related in the Implicated Site Nucleotides (ISN) of RNA interacting with the amino acids at the primitive step of life (review in [33]). According to a great number of biological experiments, the ISN structure contains nucleotides in fixed and variable positions, as well as an important trinucleotide for interacting with the amino acid (see e.g., the recent review in [34]). However, the general structure of the aptamers binding amino acids, in particular its nucleotide length, its amino acid binding loop and its nucleotide position, is still an open problem. Similar arguments could hold for the ribonucleopeptides which could be implicated in a primitive T box riboswitch functioning as an aminoacyl-tRNA synthetase and a peptidyl-transferase ribozyme [35]. The single-frame motifs SF and the framing motifs F with their properties to decode the trinucleotides and the reading frame could have been necessary for the evolutionary construction of the modern genetic code.

## Figures and Tables

**Table 1:** The 16 bidirectional multiple-frame dicodons BMF (Definition 13).

Dicodon	Frame 1	Frame 2	Dicodon	Frame 1	Frame 2	Dicodon	Frame 1	Frame 2	Dicodon	Frame 1	Frame 2
*AAAAAA*	*AAA*	*AAA*	*CACACA*	*ACA*	*CAC*	*GAGAGA*	*AGA*	*GAG*	*TATATA*	*ATA*	*TAT*
*ACACAC*	*CAC*	*ACA*	*CCCCCC*	*CCC*	*CCC*	*GCGCGC*	*CGC*	*GCG*	*TCTCTC*	*CTC*	*TCT*
*AGAGAG*	*GAG*	*AGA*	*CGCGCG*	*GCG*	*CGC*	*GGGGGG*	*GGG*	*GGG*	*TGTGTG*	*GTG*	*TGT*
*ATATAT*	*TAT*	*ATA*	*CTCTCT*	*TCT*	*CTC*	*GTGTGT*	*TGT*	*GTG*	*TTTTTT*	*TTT*	*TTT*

**Table 2:** The 96 unidirectional multiple-frame dicodons 3′UMF (Definition 11), N being any nucleotide.

Dicodon	Frame 1	Frame 2	Dicodon	Frame 1	Frame 2	Dicodon	Frame 1	Frame 2	Dicodon	Frame 1	Frame 2
*CAAAAA*	*AAA*	*AAA*	*CCACAC*	*CAC*		*CGAGAG*	*GAG*		*CTATAT*	*TAT*	
*GAAAAA*	*AAA*	*AAA*	*GCACAC*	*CAC*		*GGAGAG*	*GAG*		*GTATAT*	*TAT*	
*TAAAAA*	*AAA*	*AAA*	*TCACAC*	*CAC*		*TGAGAG*	*GAG*		*TTATAT*	*TAT*	
*NCAAAA*		*AAA*	*ACCCCC*	*CCC*	*CCC*	*AGCGCG*	*GCG*		*ATCTCT*	*TCT*	
*NGAAAA*		*AAA*	*GCCCCC*	*CCC*	*CCC*	*GGCGCG*	*GCG*		*GTCTCT*	*TCT*	
*NTAAAA*		*AAA*	*TCCCCC*	*CCC*	*CCC*	*TGCGCG*	*GCG*		*TTCTCT*	*TCT*	
*AACACA*	*ACA*		*NACCCC*		*CCC*	*AGGGGG*	*GGG*	*GGG*	*ATGTGT*	*TGT*	
*GACACA*	*ACA*		*NGCCCC*		*CCC*	*CGGGGG*	*GGG*	*GGG*	*CTGTGT*	*TGT*	
*TACACA*	*ACA*		*NTCCCC*		*CCC*	*TGGGGG*	*GGG*	*GGG*	*TTGTGT*	*TGT*	
*AAGAGA*	*AGA*		*ACGCGC*	*CGC*		*NAGGGG*		*GGG*	*ATTTTT*	*TTT*	*TTT*
*CAGAGA*	*AGA*		*CCGCGC*	*CGC*		*NCGGGG*		*GGG*	*CTTTTT*	*TTT*	*TTT*
*TAGAGA*	*AGA*		*TCGCGC*	*CGC*		*NTGGGG*		*GGG*	*GTTTTT*	*TTT*	*TTT*
*AATATA*	*ATA*		*ACTCTC*	*CTC*		*AGTGTG*	*GTG*		*NATTTT*		*TTT*
*CATATA*	*ATA*		*CCTCTC*	*CTC*		*CGTGTG*	*GTG*		*NCTTTT*		*TTT*
*GATATA*	*ATA*		*GCTCTC*	*CTC*		*GGTGTG*	*GTG*		*NGTTTT*		*TTT*

**Table 3:** The 96 unidirectional multiple-frame dicodons $5^{\prime }UMF$ (Definition 12), N being any nucleotide.

Dicodon	Frame 1	Frame 2	Dicodon	Frame 1	Frame 2	Dicodon	Frame 1	Frame 2	Dicodon	Frame 1	Frame 2
*AAAAAC*	*AAA*	*AAA*	*CACACC*		*CAC*	*GAGAGC*		*GAG*	*TATATC*		*TAT*
*AAAAAG*	*AAA*	*AAA*	*CACACG*		*CAC*	*GAGAGG*		*GAG*	*TATATG*		*TAT*
*AAAAAT*	*AAA*	*AAA*	*CACACT*		*CAC*	*GAGAGT*		*GAG*	*TATATT*		*TAT*
*AAAACN*	*AAA*		*CCCCCA*	*CCC*	*CCC*	*GCGCGA*		*GCG*	*TCTCTA*		*TCT*
*AAAAGN*	*AAA*		*CCCCCG*	*CCC*	*CCC*	*GCGCGG*		*GCG*	*TCTCTG*		*TCT*
*AAAATN*	*AAA*		*CCCCCT*	*CCC*	*CCC*	*GCGCGT*		*GCG*	*TCTCTT*		*TCT*
*ACACAA*		*ACA*	*CCCCAN*	*CCC*		*GGGGGA*	*GGG*	*GGG*	*TGTGTA*		*TGT*
*ACACAG*		*ACA*	*CCCCGN*	*CCC*		*GGGGGC*	*GGG*	*GGG*	*TGTGTC*		*TGT*
*ACACAT*		*ACA*	*CCCCTN*	*CCC*		*GGGGGT*	*GGG*	*GGG*	*TGTGTT*		*TGT*
*AGAGAA*		*AGA*	*CGCGCA*		*CGC*	*GGGGAN*	*GGG*		*TTTTTA*	*TTT*	*TTT*
*AGAGAC*		*AGA*	*CGCGCC*		*CGC*	*GGGGCN*	*GGG*		*TTTTTC*	*TTT*	*TTT*
*AGAGAT*		*AGA*	*CGCGCT*		*CGC*	*GGGGTN*	*GGG*		*TTTTTG*	*TTT*	*TTT*
*ATATAA*		*ATA*	*CTCTCA*		*CTC*	*GTGTGA*		*GTG*	*TTTTAN*	*TTT*	
*ATATAC*		*ATA*	*CTCTCC*		*CTC*	*GTGTGC*		*GTG*	*TTTTCN*	*TTT*	
*ATATAG*		*ATA*	*CTCTCG*		*CTC*	*GTGTGG*		*GTG*	*TTTTGN*	*TTT*	

**Table 4:** Probability PbSFM(n) (%) of single-frame n -motifs SF for n∈{1,…,6}. Exact and simulated values of PbSFM(n) are identical at $10^{-3}$.

		Probability PbSFM(n) (%)	
n-Motifs	$\mathrm{Number}\;64^{n}$	Exact Values	Simulated Values
1	64	100	
2	4096	94.92	94.93
3	262,144	85.22	85.20
4	16,777,216	72.35	72.37
5	1,073,741,824	58.07	58.08
6	68,719,476,736	44.07	44.08

**Table 5:** The 16 BMF dipeptides coded by the 16 bidirectional multiple-frame dicodons BMF (Definition 13, Table 1).

*AR*	*AlaArg*	*GCGCGC*	*GG*	*GlyGly*	*GGGGGG*	*LS*	*LeuSer*	*CTCTCT*	*SL*	*SerLeu*	*TCTCTC*
*CV*	*CysVal*	*TGTGTG*	*HT*	*HisThr*	*CACACA*	*PP*	*ProPro*	*CCCCCC*	*TH*	*ThrHis*	*ACACAC*
*ER*	*GluArg*	*GAGAGA*	*IY*	*IleTyr*	*ATATAT*	*RA*	*ArgAla*	*CGCGCG*	*VC*	*ValCys*	*GTGTGT*
*FF*	*PhePhe*	*TTTTTT*	*KK*	*LysLys*	*AAAAAA*	*RE*	*ArgGlu*	*AGAGAG*	*YI*	*TyrIle*	*TATATA*

**Table 6:** Occurrence number of the 15 amino acids in the 1st and 2nd positions of the 16 BMF dipeptides (Table 5).

	*A*	*C*	*E*	*F*	*G*	*H*	*I*	*K*	*L*	*P*	*R*	*S*	*T*	*V*	*Y*	
	*Ala*	*Cys*	*Glu*	*Phe*	*Gly*	*His*	*Ile*	*Lys*	*Leu*	*Pro*	*Arg*	*Ser*	*Thr*	*Val*	*Tyr*	Sum
1st site	1	1	1	1	1	1	1	1	1	1	2	1	1	1	1	16
2nd site	1	1	1	1	1	1	1	1	1	1	2	1	1	1	1	16
Sum	2	2	2	2	2	2	2	2	2	2	4	2	2	2	2	32

**Table 7:** The 83 3′UMF dipeptides and the four pairs (stop codon, amino acid) coded by the 96 unidirectional multiple-frame dicodons 3′UMF (Definition 11, Table 2).

*AF*	*AlaPhe*	*GCTTTT*	*IS*	*IleSer*	*ATCTCT*	*RV*	*ArgVal*	*CGTGTG*
*AG*	*AlaGly*	*GCGGGG*	*KG*	*LysGly*	*AAGGGG*	*SA*	*SerAla*	*AGCGCG*
*AH*	*AlaHis*	*GCACAC*	*KR*	*LysArg*	*AAGAGA*	*SF*	*SerPhe*	*AGTTTT, TCTTTT*
*AK*	*AlaLys*	*GCAAAA*	*LC*	*LeuCys*	*CTGTGT, TTGTGT*	*SG*	*SerGly*	*TCGGGG*
*AL*	*AlaLeu*	*GCTCTC*	*LF*	*LeuPhe*	*CTTTTT*	*SH*	*SerHis*	*TCACAC*
*AP*	*AlaPro*	*GCCCCC*	*LG*	*LeuGly*	*CTGGGG, TTGGGG*	*SK*	*SerLys*	*TCAAAA*
*CA*	*CysAla*	*TGCGCG*	*LK*	*LeuLys*	*CTAAAA, TTAAAA*	*SP*	*SerPro*	*AGCCCC, TCCCCC*
*CF*	*CysPhe*	*TGTTTT*	*LP*	*LeuPro*	*CTCCCC*	*SR*	*SerArg*	*TCGCGC*
*CP*	*CysPro*	*TGCCCC*	*LY*	*LeuTyr*	*CTATAT, TTATAT*	*SV*	*SerVal*	*AGTGTG*
*DF*	*AspPhe*	*GATTTT*	*MC*	*MetCys*	*ATGTGT*	*TerE*	*TerGlu*	*TGAGAG*
*DI*	*AspIle*	*GATATA*	*MG*	*MetGly*	*ATGGGG*	*TerG*	*TerGly*	*TAGGGG*
*DP*	*AspPro*	*GACCCC*	*NF*	*AsnPhe*	*AATTTT*	*TerK*	*TerLys*	*TAAAAA, TGAAAA*
*DT*	*AspThr*	*GACACA*	*NI*	*AsnIle*	*AATATA*	*TerR*	*TerArg*	*TAGAGA*
*EG*	*GluGly*	*GAGGGG*	*NP*	*AsnPro*	*AACCCC*	*TF*	*ThrPhe*	*ACTTTT*
*EK*	*GluLys*	*GAAAAA*	*NT*	*AsnThr*	*AACACA*	*TG*	*ThrGly*	*ACGGGG*
*FP*	*PhePro*	*TTCCCC*	*PF*	*ProPhe*	*CCTTTT*	*TK*	*ThrLys*	*ACAAAA*
*FS*	*PheSer*	*TTCTCT*	*PG*	*ProGly*	*CCGGGG*	*TL*	*ThrLeu*	*ACTCTC*
*GA*	*GlyAla*	*GGCGCG*	*PH*	*ProHis*	*CCACAC*	*TP*	*ThrPro*	*ACCCCC*
*GE*	*GlyGlu*	*GGAGAG*	*PK*	*ProLys*	*CCAAAA*	*TR*	*ThrArg*	*ACGCGC*
*GF*	*GlyPhe*	*GGTTTT*	*PL*	*ProLeu*	*CCTCTC*	*VF*	*ValPhe*	*GTTTTT*
*GK*	*GlyLys*	*GGAAAA*	*PR*	*ProArg*	*CCGCGC*	*VG*	*ValGly*	*GTGGGG*
*GP*	*GlyPro*	*GGCCCC*	*QG*	*GlnGly*	*CAGGGG*	*VK*	*ValLys*	*GTAAAA*
*GV*	*GlyVal*	*GGTGTG*	*QK*	*GlnLys*	*CAAAAA*	*VP*	*ValPro*	*GTCCCC*
*HF*	*HisPhe*	*CATTTT*	*QR*	*GlnArg*	*CAGAGA*	*VS*	*ValSer*	*GTCTCT*
*HI*	*HisIle*	*CATATA*	*RE*	*ArgGlu*	*CGAGAG*	*VY*	*ValTyr*	*GTATAT*
*HP*	*HisPro*	*CACCCC*	*RF*	*ArgPhe*	*CGTTTT*	*WG*	*TrpGly*	*TGGGGG*
*IF*	*IlePhe*	*ATTTTT*	*RG*	*ArgGly*	*AGGGGG, CGGGGG*	*YF*	*TyrPhe*	*TATTTT*
*IK*	*IleLys*	*ATAAAA*	*RK*	*ArgLys*	*AGAAAA, CGAAAA*	*YP*	*TyrPro*	*TACCCC*
*IP*	*IlePro*	*ATCCCC*	*RP*	*ArgPro*	*CGCCCC*	*YT*	*TyrThr*	*TACACA*

**Table 8:** Occurrence number of the 20 amino acids in the first and second positions of the 83 3′UMF dipeptides and the four pairs (stop codon, amino acid) (Table 7).

	*A*	*C*	*D*	*E*	*F*	*G*	*H*	*I*	*K*	*L*	*M*	*N*	*P*	*Q*	*R*	*S*	*T*	*V*	*W*	*Y*		
	*Ala*	*Cys*	*Asp*	*Glu*	*Phe*	*Gly*	*His*	*Ile*	*Lys*	*Leu*	*Met*	*Asn*	*Pro*	*Gln*	*Arg*	*Ser*	*Thr*	*Val*	*Trp*	*Tyr*	*Ter*	Sum
1st site	6	3	4	2	2	6	3	4	2	6	2	4	6	3	6	8	6	6	1	3	4	87
2nd site	3	2	0	3	14	13	3	3	12	3	0	0	14	0	6	3	3	3	0	2	0	87
Sum	9	5	4	5	16	19	6	7	14	9	2	4	20	3	12	11	9	9	1	5	4	174

**Table 9:** The 40 $5^{\prime }UMF$ dipeptides and the three pairs (amino acid, stop codon) coded by the 96 unidirectional multiple-frame dicodons $5^{\prime }UMF$ (Definition 12, Table 3).

*AR*	*AlaArg*	*GCGCGA, GCGCGG, GCGCGT*	*KN*	*LysAsn*	*AAAAAC, AAAAAT*
*CV*	*CysVal*	*TGTGTA, TGTGTC, TGTGTT*	*KR*	*LysArg*	*AAAAGA, AAAAGG*
*ER*	*GluArg*	*GAGAGG*	*KS*	*LysSer*	*AAAAGC, AAAAGT*
*ES*	*GluSer*	*GAGAGC, GAGAGT*	*KT*	*LysThr*	*AAAACA, AAAACC, AAAACG, AAAACT*
*FC*	*PheCys*	*TTTTGC, TTTTGT*	*LS*	*LeuSer*	*CTCTCA, CTCTCC, CTCTCG*
*FF*	*PhePhe*	*TTTTTC*	*PH*	*ProHis*	*CCCCAC, CCCCAT*
*FL*	*PheLeu*	*TTTTTA, TTTTTG*	*PL*	*ProLeu*	*CCCCTA, CCCCTC, CCCCTG, CCCCTT*
*FS*	*PheSer*	*TTTTCA, TTTTCC, TTTTCG, TTTTCT*	*PP*	*ProPro*	*CCCCCA, CCCCCG, CCCCCT*
*FTer*	*PheTer*	*TTTTAA, TTTTAG, TTTTGA*	*PQ*	*ProGln*	*CCCCAA, CCCCAG*
*FW*	*PheTrp*	*TTTTGG*	*PR*	*ProArg*	*CCCCGA, CCCCGC, CCCCGG, CCCCGT*
*FY*	*PheTyr*	*TTTTAC, TTTTAT*	*RA*	*ArgAla*	*CGCGCA, CGCGCC, CGCGCT*
*GA*	*GlyAla*	*GGGGCA, GGGGCC, GGGGCG, GGGGCT*	*RD*	*ArgAsp*	*AGAGAC, AGAGAT*
*GD*	*GlyAsp*	*GGGGAC, GGGGAT*	*RE*	*ArgGlu*	*AGAGAA*
*GE*	*GlyGlu*	*GGGGAA, GGGGAG*	*SL*	*SerLeu*	*TCTCTA, TCTCTG, TCTCTT*
*GG*	*GlyGly*	*GGGGGA, GGGGGC, GGGGGT*	*TH*	*ThrHis*	*ACACAT*
*GV*	*GlyVal*	*GGGGTA, GGGGTC, GGGGTG, GGGGTT*	*TQ*	*ThrGln*	*ACACAA, ACACAG*
*HT*	*HisThr*	*CACACC, CACACG, CACACT*	*VC*	*ValCys*	*GTGTGC*
*ITer*	*IleTer*	*ATATAA, ATATAG*	*VTer*	*ValTer*	*GTGTGA*
*IY*	*IleTyr*	*ATATAC*	*VW*	*ValTrp*	*GTGTGG*
*KI*	*LysIle*	*AAAATA, AAAATC, AAAATT*	*YI*	*TyrIle*	*TATATC, TATATT*
*KK*	*LysLys*	*AAAAAG*	*YM*	*TyrMet*	*TATATG*
*KM*	*LysMet*	*AAAATG*			

**Table 10:** Occurrence number of the 20 amino acids in the first and second positions of the 40 $5^{\prime }UMF$ dipeptides and the three pairs (amino acid, stop codon) (Table 9).

	*A*	*C*	*D*	*E*	*F*	*G*	*H*	*I*	*K*	*L*	*M*	*N*	*P*	*Q*	*R*	*S*	*T*	*V*	*W*	*Y*		
	*Ala*	*Cys*	*Asp*	*Glu*	*Phe*	*Gly*	*His*	*Ile*	*Lys*	*Leu*	*Met*	*Asn*	*Pro*	*Gln*	*Arg*	*Ser*	*Thr*	*Val*	*Trp*	*Tyr*	*Ter*	Sum
1st site	1	1	0	2	7	5	1	2	7	1	0	0	5	0	3	1	2	3	0	2	0	43
2nd site	2	2	2	2	1	1	2	2	1	3	2	1	1	2	4	4	2	2	2	2	3	43
Sum	3	3	2	4	8	6	3	4	8	4	2	1	6	2	7	5	4	5	2	4	3	86

**Table 11:** Multi-frame dipeptide boolean matrix. The 114=121−4−3 MF dipeptides, the four pairs (stop codon, amino acid) and the three pairs (amino acid, stop codon) coded by the 208=16+2×96 multiple-frame dicodons BMF (Definition 13, Table 1), 3′UMF (Definition 11, Table 2) and $5^{\prime }UMF$ (Definition 12, Table 3). The rows and columns are associated with the first and second amino acid, respectively, in the dipeptide. The value of 1 means a MF dipeptide coded by at least a multiple-frame dicodon MF (MF true). The value of 0 stands for a SF dipeptide coded by a single-frame dicodon SF (MF false). For example, the value of *AlaCys* is 0 (absent in Table 5, Table 7 and Table 9) and the value of *CysAla* is 1 (7th row in Table 7).

Site	2nd	*A*	*C*	*D*	*E*	*F*	*G*	*H*	*I*	*K*	*L*	*M*	*N*	*P*	*Q*	*R*	*S*	*T*	*V*	*W*	*Y*		
1st		*Ala*	*Cys*	*Asp*	*Glu*	*Phe*	*Gly*	*His*	*Ile*	*Lys*	*Leu*	*Met*	*Asn*	*Pro*	*Gln*	*Arg*	*Ser*	*Thr*	*Val*	*Trp*	*Tyr*	*Ter*	Sum
*A*	*Ala*	0	0	0	0	1	1	1	0	1	1	0	0	1	0	1	0	0	0	0	0	0	7
*C*	*Cys*	1	0	0	0	1	0	0	0	0	0	0	0	1	0	0	0	0	1	0	0	0	4
*D*	*Asp*	0	0	0	0	1	0	0	1	0	0	0	0	1	0	0	0	1	0	0	0	0	4
*E*	*Glu*	0	0	0	0	0	1	0	0	1	0	0	0	0	0	1	1	0	0	0	0	0	4
*F*	*Phe*	0	1	0	0	1	0	0	0	0	1	0	0	1	0	0	1	0	0	1	1	1	8
*G*	*Gly*	1	0	1	1	1	1	0	0	1	0	0	0	1	0	0	0	0	1	0	0	0	8
*H*	*His*	0	0	0	0	1	0	0	1	0	0	0	0	1	0	0	0	1	0	0	0	0	4
*I*	*Ile*	0	0	0	0	1	0	0	0	1	0	0	0	1	0	0	1	0	0	0	1	1	6
*K*	*Lys*	0	0	0	0	0	1	0	1	1	0	1	1	0	0	1	1	1	0	0	0	0	8
*L*	*Leu*	0	1	0	0	1	1	0	0	1	0	0	0	1	0	0	1	0	0	0	1	0	7
*M*	*Met*	0	1	0	0	0	1	0	0	0	0	0	0	0	0	0	0	0	0	0	0	0	2
*N*	*Asn*	0	0	0	0	1	0	0	1	0	0	0	0	1	0	0	0	1	0	0	0	0	4
*P*	*Pro*	0	0	0	0	1	1	1	0	1	1	0	0	1	1	1	0	0	0	0	0	0	8
*Q*	*Gln*	0	0	0	0	0	1	0	0	1	0	0	0	0	0	1	0	0	0	0	0	0	3
*R*	*Arg*	1	0	1	1	1	1	0	0	1	0	0	0	1	0	0	0	0	1	0	0	0	8
*S*	*Ser*	1	0	0	0	1	1	1	0	1	1	0	0	1	0	1	0	0	1	0	0	0	9
*T*	*Thr*	0	0	0	0	1	1	1	0	1	1	0	0	1	1	1	0	0	0	0	0	0	8
*V*	*Val*	0	1	0	0	1	1	0	0	1	0	0	0	1	0	0	1	0	0	1	1	1	9
*W*	*Trp*	0	0	0	0	0	1	0	0	0	0	0	0	0	0	0	0	0	0	0	0	0	1
*Y*	*Tyr*	0	0	0	0	1	0	0	1	0	0	1	0	1	0	0	0	1	0	0	0	0	5
	*Ter*	0	0	0	1	0	1	0	0	1	0	0	0	0	0	1	0	0	0	0	0	0	4
	Sum	4	4	2	3	15	14	4	5	13	5	2	1	15	2	8	6	5	4	2	4	3	121

**Table 12:** Multi-frame dipeptide occurrence matrix. The 114=121−4−3 MF dipeptides, the four pairs (stop codon, amino acid) and the three pairs (amino acid, stop codon) coded by the 208=16+2×96 multiple-frame dicodons BMF (Definition 13, Table 1), 3′UMF (Definition 11, Table 2) and $5^{\prime }UMF$ (Definition 12, Table 3). The rows and columns are associated with the first and second amino acid, respectively, in the dipeptide. The values between 1 and 5 give the number of times a MF dipeptide is coded by multiple-frame dicodons MF. The value of 0 stands for a SF dipeptide coded by a single-frame dicodon SF. For example, the value of *AlaCys* is 0 (absent in Table 5, Table 7 and Table 9), the value of *CysAla* is 1 (7th row in Table 7) and the value of *AlaArg* if 4 (one occurrence: 1st row in Table 5 and three occurrences: 1st row in Table 9).

Site	2nd	*A*	*C*	*D*	*E*	*F*	*G*	*H*	*I*	*K*	*L*	*M*	*N*	*P*	*Q*	*R*	*S*	*T*	*V*	*W*	*Y*		
1st		*Ala*	*Cys*	*Asp*	*Glu*	*Phe*	*Gly*	*His*	*Ile*	*Lys*	*Leu*	*Met*	*Asn*	*Pro*	*Gln*	*Arg*	*Ser*	*Thr*	*Val*	*Trp*	*Tyr*	*Ter*	Sum
*A*	*Ala*	0	0	0	0	1	1	1	0	1	1	0	0	1	0	4	0	0	0	0	0	0	10
*C*	*Cys*	1	0	0	0	1	0	0	0	0	0	0	0	1	0	0	0	0	4	0	0	0	7
*D*	*Asp*	0	0	0	0	1	0	0	1	0	0	0	0	1	0	0	0	1	0	0	0	0	4
*E*	*Glu*	0	0	0	0	0	1	0	0	1	0	0	0	0	0	2	2	0	0	0	0	0	6
*F*	*Phe*	0	2	0	0	2	0	0	0	0	2	0	0	1	0	0	5	0	0	1	2	3	18
*G*	*Gly*	5	0	2	3	1	4	0	0	1	0	0	0	1	0	0	0	0	5	0	0	0	22
*H*	*His*	0	0	0	0	1	0	0	1	0	0	0	0	1	0	0	0	4	0	0	0	0	7
*I*	*Ile*	0	0	0	0	1	0	0	0	1	0	0	0	1	0	0	1	0	0	0	2	2	8
*K*	*Lys*	0	0	0	0	0	1	0	3	2	0	1	2	0	0	3	2	4	0	0	0	0	18
*L*	*Leu*	0	2	0	0	1	2	0	0	2	0	0	0	1	0	0	4	0	0	0	2	0	14
*M*	*Met*	0	1	0	0	0	1	0	0	0	0	0	0	0	0	0	0	0	0	0	0	0	2
*N*	*Asn*	0	0	0	0	1	0	0	1	0	0	0	0	1	0	0	0	1	0	0	0	0	4
*P*	*Pro*	0	0	0	0	1	1	3	0	1	5	0	0	4	2	5	0	0	0	0	0	0	22
*Q*	*Gln*	0	0	0	0	0	1	0	0	1	0	0	0	0	0	1	0	0	0	0	0	0	3
*R*	*Arg*	4	0	2	3	1	2	0	0	2	0	0	0	1	0	0	0	0	1	0	0	0	16
*S*	*Ser*	1	0	0	0	2	1	1	0	1	4	0	0	2	0	1	0	0	1	0	0	0	14
*T*	*Thr*	0	0	0	0	1	1	2	0	1	1	0	0	1	2	1	0	0	0	0	0	0	10
*V*	*Val*	0	2	0	0	1	1	0	0	1	0	0	0	1	0	0	1	0	0	1	1	1	10
*W*	*Trp*	0	0	0	0	0	1	0	0	0	0	0	0	0	0	0	0	0	0	0	0	0	1
*Y*	*Tyr*	0	0	0	0	1	0	0	3	0	0	1	0	1	0	0	0	1	0	0	0	0	7
	*Ter*	0	0	0	1	0	1	0	0	2	0	0	0	0	0	1	0	0	0	0	0	0	5
	Sum	11	7	4	7	17	19	7	9	17	13	2	2	19	4	18	15	11	11	2	7	6	208

## Abbreviations

SF	single-frame motif (unambiguous trinucleotide decoding in the two $5^{\prime }–3^{\prime }$ and $3^{\prime }–5\prime$ directions)
MF	multiple-frame motif
UMF	unidirectional multiple-frame motif
$3^{\prime }UMF$	unidirectional multiple-frame motif (ambiguous trinucleotide decoding in the $3^{\prime }–5^{\prime }$ direction only)
$5^{\prime }UMF$	unidirectional multiple-frame motif (ambiguous trinucleotide decoding in the $5^{\prime }–3^{\prime }$ direction only)
BMF	bidirectional multiple-frame motif (ambiguous trinucleotide decoding in the two $5^{\prime }–3^{\prime }$ and $3^{\prime }–5^{\prime }$ directions)
$5^{\prime }U$	5′ unambiguous motif (unambiguous trinucleotide decoding in the $5^{\prime }–3^{\prime }$ direction only)
F	framing motif (also called circular code motif)
